# Human resource leadership: the key to improved results in health

**DOI:** 10.1186/1478-4491-6-10

**Published:** 2008-06-20

**Authors:** Mary L O'Neil

**Affiliations:** 1 Center for Leadership and Management, Management Sciences for Health, Cambridge, MA, USA

## Abstract

This article is the lead article in the Human Resources for Health journal's first quarterly feature. The series of seven articles has been contributed by Management Sciences for Health (MSH) under the theme of leadership and management in public health and will be published article by article over the next few weeks. The journal has invited Dr Manuel M. Dayrit, Director of the WHO Department of Human Resources for Health and former Minister of Health for the Philippines to launch the feature with an opening editorial to be found in the journal's blog.

This opening article describes the human resource challenges that managers around the world report and analyses why solutions often fail to be implemented.

Despite rising attention to the acute shortage of health care workers, solutions to the human resource (HR) crisis are difficult to achieve, especially in the poorest countries. Although we are aware of the issues and have developed HR strategies, the problem is that some old systems of leading and managing human resources for health do not work in today's context.

The Leadership Development Program (LDP) is grounded on the belief that good leadership and management can be learned and practiced at all levels. The case studies in this issue were chosen to illustrate results from using the LDP at different levels of the health sector.

The LDP makes a profound difference in health managers' attitudes towards their work. Rather than feeling defeated by a workplace climate that lacks motivation, hope, and commitment to change, people report that they are mobilized to take action to change the status quo. The lesson is that without this capacity at all levels, global policy and national HR strategies will fail to make a difference.

## Background

Despite rising attention to the acute shortage of health care workers, solutions to the human resource (HR) crisis are difficult to achieve, especially in the poorest countries. Although we are aware of the issues and have developed HR strategies, the problem is that some old systems of leading and managing human resources for health do not work in today's context. In these cases and others, a more appropriate mode of leadership, linked to reforming management systems and committed to moving beyond planning to implementation, is essential to the solution.

**Figure 1:**
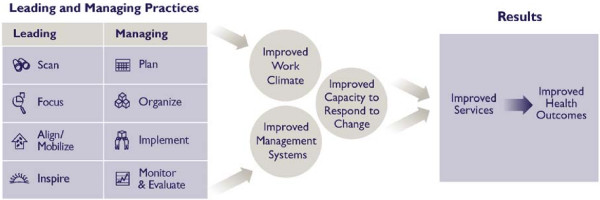
Leading and managing results model – how do management and leadership contribute to improved service delivery?

The Leadership Development Program (LDP), based on the model shown in Figure 1, is grounded on the belief that good leadership and management can be learned and practiced at all levels. The principles of this approach are:

▪ focusing on health outcomes

▪ working in teams to solve problems

▪ applying leadership practices to real challenges

▪ creating a climate that encourages change

▪ sustaining change by grounding solutions in management systems.

The case studies in this issue were chosen to illustrate results from using the LDP at different levels of the health sector:

▪ Central level: emergency hiring program in Kenya.

▪ Local level: empowering workers in Mozambique.

▪ Civil society: improving retention and performance in Uganda.

The format of the LDP has been adapted, for example, to accommodate people from rural areas. The program has been translated into French, Spanish, Portuguese, and Arabic. One important finding is that participants often replicate the principles in many areas of their work and teach them to others.

The LDP makes a profound difference in health managers' attitudes toward their work. Rather than feeling defeated by a workplace climate that lacks motivation, hope, and commitment to change, people report that they are mobilized to take action to change the status quo. The lesson is that without this capacity at all levels, global policy and national HR strategies will fail to make a difference.

## Discussion

The dimensions of the HR crisis in health have been reported in stark terms in publications and studies for years by the Joint Learning Initiative [1], the World Health Organization (WHO) [2], and others [3]. With the formation of the Global Health Workforce Alliance and the commitment of organizations such as the WHO, we now have mechanisms to provide leadership at the global level. But strategic HR leadership (managing people as a strategic resource) poses a significant challenge for most ministries of health because HR management policies and practices are not in professional hands. Governments lack the ability to adapt to rapid changes such as labor migration, the impact of HIV/AIDS, structural adjustments and hiring freezes. Countries need visionary leaders to advocate that funding for HR solutions go hand-in-hand with funding for health programs. Millions of dollars, for example, have been invested to ensure the availability of AIDS and tuberculosis drugs, but hardly any funding has been committed to ensure that there is a sustainable workforce to administer these drugs.

At all levels of the health system, what is lacking is a critical mass of proactive and respected HR managers and specialists who are professionally qualified and have the authority and clout to attract attention and deal with these challenges and champion a comprehensive response. To make significant improvements in human resources for health and in the health of populations – improvements that will last – managers need to know how to lead and how to influence HR changes within and outside their organizations.

### Human resource challenges reported by leaders and managers

Health leaders and managers report that they are mired in layers of civil service rules, highly centralized and fragmented HR management systems, poor incentives, underuse or misuse of existing staff, and external pressure to diminish social-sector expenditure. Managers in many countries say that the leadership to address these issues is lacking. There are pilot projects and examples of effective HR solutions at the country level, but HR directors and policymakers need a new way of thinking about leading and managing to sustain these positive results and scale them up. Health managers at a recent HR leadership meeting in Southern Africa described their problems as follows: "We are certainly weakest at implementing. We agree on weekly, monthly, and quarterly activities – but at the end of the day almost nothing gets done...."

These sentiments are echoed around the world. Most countries have clearly identified the HR challenges they face and many have developed an HR strategy, but these often stay on the shelf and are not implemented in any systematic way that will achieve their intended outcomes. Often the reason cited for this failure is lack of funding, but we propose that, even with adequate funding, efforts to implement HR plans and obtain results may fail due to other factors.

### Factors that contribute to the failed implementation of human resource strategies

#### ▪ Fragmentation of effort

Donors fund pieces of the solution, with little harmonization among these partial approaches, diminishing the impact that could result from a comprehensive, harmonized approach.

#### ▪ Unrealistic time frames

HR solutions require time (at least three to five years) to produce results.

#### ▪ No grounding in management systems

HR interventions are often not grounded in any sustainable HR management (HRM) system. HRM systems are fragmented, with authority for HR planning, recruitment, hiring, deployment, promotion, compensation, incentives, and staff development spread among several ministries. These systems need to be streamlined and staffed by trained HR professionals before lasting change can occur.

#### ▪ Key stakeholders, especially outside the health sector, are not involved

Ministries of health cannot solve the HR crisis on their own. Ministries of civil service, finance, and education, and the private sector all have a role to play.

Progress in dealing with these factors depends on managers who are able to lead and inspire teams at all levels of the health system to transform strategies, plans, and recommendations into a comprehensive and harmonized approach. At present, most government HR functions are handled by personnel administrators who were trained to handle routine civil service policies and procedures. There is an urgent need to professionalize this cadre, expand the organizational view of their roles, and update their skills so they can be more effective in leading change and implementing plans.

This is not the traditional approach to developing leadership, which is aimed at top leaders and focuses on leadership traits and characteristics. This model focuses on developing teams that can identify problems, find solutions, and get results (see Figure 1).

### Case studies in applying leadership practices

The three articles that follow in this series constituting the special quarterly feature on leadership illustrate what can happen when managers put the MSH model into practice. We offer examples of HR activities carried out in the public sector at both the central and district levels, as well as an example from civil society.

## Conclusion

The dialogue about human resources for health today is broad. It offers data on the shortages of health workers and the human toll that results, it provides a detailed analysis of the causes of the crisis, and it presents a wide range of solutions from task-shifting to new models for community nursing. It asks tough questions about whether International Monetary Fund policies constrain health spending in poor countries, about the consequences of a health strategy that is built around a 50% focus on HIV/AIDS, and about the responsibility of wealthy countries recruiting needed health staff from the poorest countries in the world. The dialogue is also asking questions about the best way to move from awareness and analysis to action. The answers to this question are many and depend on the situation of each country and its strengths and weaknesses.

One imperative for all countries, however, is the leadership and management capability to translate HR strategies into systems and practices that result in sustainable improvements. At the beginning of this piece, HR managers who were uninspired concerning their ability to bring about these kinds of positive changes were cited. HR managers who participated in a Leadership Development Program noted a profound difference: "We had discussed the challenges before, but the leadership program actually got us mobilized to complete it."

The conclusion? The complex conditions under which we work to address the HR crisis demand a new style of leadership that encourages innovation and teamwork and a more professional approach to HR management. At all levels we need committed leaders and qualified HR managers to translate country-level strategies and global frameworks for migration, financing options, and fast-tracking of education and HR reform into solutions on the ground.

## Competing interests

The author declares that she has no competing interests.

## Authors' contributions

Dr MO researched and wrote this article and read and approved the final manuscript.
